# Garcinone C inhibits pseudorabies virus replication through EGF/PI3K/Akt axis

**DOI:** 10.3389/fcimb.2025.1722752

**Published:** 2026-02-02

**Authors:** Changjie Lv, Shuang Wang, Zhongyuan Jin, Jiaxin Zhang, Yajie Peng, Jinmiao Chen

**Affiliations:** 1 Key Laboratory of Animal Pathogen Infection and Immunology of Fujian Province, College of Animal Sciences, Fujian Agriculture and Forestry University, Fuzhou, China; 2 Joint Laboratory of Animal Pathogen Prevention and Control of Fujian-Nepal, College of Animal Sciences, Fujian Agriculture and Forestry University, Fuzhou, China; 3 Key Laboratory of Fujian-Taiwan Animal Pathogen Biology, College of Animal Sciences, Fujian Agriculture and Forestry University, Fuzhou, China; 4 Engineering Research Center for Animal Breeding and Sustainable Production, College of Animal Sciences, Fujian Agriculture and Forestry University, Fuzhou, China

**Keywords:** EGF, garcinone C, inflammatory process, PI3K/Akt, pseudorabies virus

## Abstract

**Introduction:**

Pseudorabies virus (PRV), a significant pathogen of swine, causes substantial economic losses, and even poses an emergent public health concern due to its zoonotic potential. The continuous evolution of PRV has undermined the effectiveness of current vaccines and antiviral drugs. Consequently, there is an urgent need to develop novel strategies to curb its spread.

**Methods:**

The inhibitory effect of garcinone C on PRV replication was assessed *in vivo* and *in vitro*. To determine the stage of antiviral action, treatments were administered at different time points: pre-treatment, co-treatment, and post-infection. RNA sequencing was performed, and differentially expressed genes (DEGs) were identified.

**Results:**

In the study, we found that garcinone C inhibited PRV replication in a concentration- and timedependent manner in vitro. The antiviral activity of garcinone C was specific to post-infection administration and did not extend to pre-treatment and cotreatment conditions. Transcriptomic analysis identified DEGs between garcinone C- and vehicle-treated cells after PRV infection. KEGG pathway enrichment analysis of the DEGs indicated that the antiviral effect of garcinone C was primarily associated with the PI3K-Akt signaling pathway, potentially through the downregulation of the host epidermal growth factor. Furthermore, garcinone C suppressed the production of key inflammatory cytokines such as IL-6, IL-8, and TNF-a during PRV infection. Oral administration of garcinone C conferred protection in PRVinfected mice, leading to attenuated weight loss, an improved survival rate, as well as reduced pathological changes and viral loads in tissues.

**Discussion:**

Collectively, our findings identify garcinone C as a promising therapeutic candidate against PRV and elucidate its underlying molecular mechanism.

## Introduction

Pseudorabies virus (PRV), a member of the *Alphaherpesvirinae* subfamily, causes Aujeszky’s disease in pigs, which can manifest as respiratory distress, nervous, and genital disorders (Ling et al., 2025; Wang et al., 2025b). The domestic pig serves as the natural reservoir and primary host for PRV, however, the virus exhibits a broad-spectrum host range, infecting diverse species such as rabbit, rodents, and guinea pig (Liu et al., 2024). Prior to 2011, pseudorabies was successfully controlled in China through the extensive application of the Bartha-K61 vaccine (Bo and X, 2022; Tan et al., 2021). However, since late 2011, frequent and rapidly spreading PR outbreaks have been reported, driven by the emergence of PRV variants (Li et al., 2017). These variants are characterized by amino acid deletions and insertions in key glycoproteins (An et al., 2013; Jiang et al., 2024). Owing to its substantial impact and severity, effective control of PRV necessitates an integrated strategy, encompassing robust vaccination programs, strict biosecurity protocols, and active surveillance systems (Fan et al., 2023; Sun et al., 2016). Recently, the isolation of a PRV strain from a case of acute human encephalitis highlights a significant public health threat, confirming the potential for cross-species transmission from swine to humans (Liu Q. et al., 2021). Consequently, there is a critical need for novel strategies to prevent and control PRV infection.

The genome of PRV is approximately 143 kb in length, which harbors roughly 70 open reading frames collectively encoding no fewer than 70 proteins (Bo et al., 2020; Zhou et al., 2025). To complete their replication cycle, these viral proteins depend on the host’s transcription and translation machinery (Wang et al., 2025c; Wu et al., 2024). Accordingly, vital host proteins involved in these processes represent potential therapeutic targets for antiviral strategies. Epidermal growth factor (EGF), a critical host protein, activates downstream signaling pathway including PI3K-Akt affecting the NF-κB signaling pathway, ultimately driving the expression of inflammatory factors (Yan et al., 2022; Yao et al., 2025). The hyperinflammatory response and subsequent multi-tissue injury driven by PRV infection are alleviated by gasdermin D deficiency, which reduces TNF-α production and viral pathogenicity (Chen et al., 2025). Inhibition of nucleotide-binding oligomerization domain 1 suppressed the activation of the NF-κB and MAPK signaling pathways in the brain tissues of PRV-infected mice (Sun et al., 2024). PRV VP22 protein interacts with host ZBP1, which blocks the recruitment of recruitment of receptor-interacting protein kinase 3 and caspase-8, ultimately suppressing NLRP3 inflammatory response (Ma et al., 2024). Thus, the host inflammatory response is a critical process of PRV proliferation, and suppressing excessive inflammation represents a promising therapeutic strategy for controlling PRV infection.

Natural products have been widely used in antiviral research (Liu X. et al., 2021). Garcinone C, a natural xanthone derivative, is isolated in the pericarp of *Garcinia mangostana* (Chen et al., 2020). Garcinone C significantly inhibits RANKL-induced ROS production and NF-κB activity, through inhibition of p65 phosphorylation as well as the phosphorylation-dependent degradation of IκBα (Ji et al., 2024). Garcinone C upregulates the expression of ATR and 4E-BP1, while effectively downregulates cyclin B1, cyclin D1, cyclin E2, cdc2, CDK7, and Stat3 in nasopharyngeal carcinoma cells (Xia et al., 2018). However, the role of garcinone C in regulating the process of PRV infection is unknown.

We found that garcinone C significantly inhibited PRV replication *in vitro* and *in vivo*. The compound exhibited a potent antiviral activity at post-infection stage. Transcriptome analysis revealed garcinone C acted by downregulating EGF, leading to the modulation of the PI3K-Akt signaling pathway. Furthermore, garcinone C decreased expression of inflammatory factors including IL-6, IL-8, and TNF-α. These findings highlight the potential of garcinone C for novel anti-PRV drug.

## Materials and methods

### Cells and virus

PK-15 cells were cultured in DMEM culture medium (Gibco, Waltham, MA, USA) supplemented with 10% fetal bovine serum (Vazyme, Nanjing, China), 100 μg/mL streptomycin, and 100 IU/mL penicillin at 37 °C in a 5% CO_2_ atmosphere. The PRV (SDFB) and swine influenza A virus H3N2 (HZNL) were isolated from Shandong, China. The GenBank accession numbers for PRV (SDFB) and H3N2 (HZNL) are PX712891 and PX705906, respectively.

### Antibodies and reagents

Antibodies of PRV gB were kept in our laboratory and used at a dilution of 1:5,000. The GAPDH monoclonal antibody was purchased from Proteintech (catalog no. 60004-1-Ig; Wuhan, China) and applied at a 1:5,000 dilution. Secondary antibodies including HRP-conjugated goat anti-mouse IgG (H+L) were obtained from Proteintech (catalog no. SA00001-1; Wuhan, China) and performed at a dilution of 1:10,000. Garcinone C were purchased from MCE (Shanghai, China) and dissolved in DMSO.

### Western blotting

The proteins were separated by electrophoresis using a 12.5% SDS-PAGE kit (Vazyme, Nanjing, China), and subsequently transferred onto a nitrocellulose membrane (Waukesha, USA). The membrane was then blocked for 1 h at 37°C with 5% non-fat milk prepared in Tris-buffered saline containing Tween-20 (TBST). After blocking, the membrane was incubated with the corresponding primary antibody at 4°C overnight. After five washes with TBST, the membrane was incubated with an HRP-conjugated secondary antibody for 1 h at 37°C. Following another five TBST washes, the protein bands were visualized using the Tanon-5200 imaging system with an ECL reagent (NCM Biotech, Suzhou, China).

### TCID_50_

PK-15 cells were cultured in 96-well plates, and inoculated with serial 10-fold dilutions of the viral stock with eight replicates per dilution. The cytopathic effect (CPE) was observed and recorded for 72 h, and the results were calculated by the Reed-Muench method.

### RNA extraction and qPCR

The total RNA was extracted using the total RNA isolation kit from Magen (Guangzhou, China) following the manufacturer’s instructions. Total RNA samples were reverse transcribed into cDNA using the reverse transcription kit (Vazyme, Nanjing, China). The synthesized cDNA served as the template for qPCR, mRNA expression levels of target genes were quantified using the QuantStudioTM 1 Plus Real-Time PCR System (Applied BiosystemsTM, Waltham, MA, USA). *GAPDH* served as the endogenous reference control for data normalization. Relative expression levels of target genes were determined using the 2^-ΔΔCT^ method.

### Cell viability assay

Cell viability was evaluated using the Cell Counting Kit-8 (GLPbio, California, USA) according to the manufacturer’s protocol. Briefly, PK-15 cells were seeded in 96-well plates, and treated with garcinone C of different concentrations for 24 h. Thereafter, 10 μL CCK-8 reagent was added to each well, and the plates were incubated at 37°C for 1 h. The absorbance of each well at 450 nm was measured using a microplate reader.

### The antiviral effect of garcinone C was assessed *in vitro*

To evaluate the efficacy of garcinone C in inhibiting PRV replication, drugs of different concentrations were used. PK-15 cells were pre-treated with varying concentrations of garcinone C or 20 μM garcinone C for 1 h prior to infection. The drug-containing medium was then removed, and the cells were inoculated with 0.05 MOI PRV for 1 h. Following the removal of the viral inoculum, the cells were washed three times with phosphate buffered saline (PBS). Fresh medium containing the respective concentrations of garcinone C was added. Finally, samples were collected at 24 hpi or different time points for analysis by Western blotting, qPCR, and TCID_50_ assay.

To determine the stage at which garcinone C exerts its antiviral activity, the compound was administered at three different stages relative to PRV infection including pre-infection, co-infection, and post-infection. At the pre-infection stage, cells were incubated with garcinone C for 1 h prior to viral infection. At the co-infection stage, garcinone C was added with PRV in cells for 1 h. At the post-infection stage, cells were treated with garcinone C after PRV infection for 1 h. In the above three processes, the viral inoculum was removed after 1 h, and the cells were washed three times with PBS. The antiviral effects of garcinone C were evaluated using Western blotting, qPCR, and TCID_50_ assays at 24 hpi.

### RNA sequencing

For transcriptomic analysis, RNA was extracted from PK-15 cells treated with garcinone C, garcinone C after PRV infection, vehicle after PRV infection, and vehicle. The quality and integrity of extracted total RNA were analyzed using 1% (wt/vol) agarose gel and a Nanodrop 2000C system (Thermo Fisher Scientific, Waltham, MA, USA). All RNA samples were delivered to Biomarker (Beijing, China) for further transcriptome sequencing. The RNA data were clustered for bioinformatic analysis by gene ontology (GO) and Kyoto Encyclopedia of Genes and Genomes (KEGG) pathway enrichment analyses.

### Animal experiments

All animal-related procedures strictly adhered to the Care and Use of Laboratory Animals guidelines approved by the Research Ethics Committee of Fujian Agriculture and Forestry University, Fujian, China. To assess the anti-PRV efficacy and safety of garcinone C, an animal study was conducted using mice *in vivo*. Female BALB/c mice aged 6–8 weeks were utilized. For the toxicity assessment of garcinone C, the mice were orally administered with garcinone C of different concentrations including 1, 5, and 10 mg/kg for 5 times, while those in control group received vehicle. The nontoxic concentration of garcinone C was determined based on the survival rates and body weight changes of the mice. To evaluate anti-PRV activity of garcinone C, mouse was intraperitoneally infected with 500 PFU PRV on 0 day, and mice were orally administered with 10 mg/kg garcinone C every other day, beginning 1 day before infection and continuing until 10 days post infection (dpi). To evaluate the anti-PRV activity of garcinone C, mice were intraperitoneally infected with 500 PFU PRV on day 0. The mice were orally administered garcinone C (10 mg/kg) every other day, from 1 day before infection until 10 days post-infection (dpi). Spleens and kidneys were harvested at 3 dpi, and viral load was detected using qPCR assay.

### Hematoxylin-eosin staining

For histopathological assessment of spleens and kidneys, hematoxylin-eosin staining was conducted according to previous research (Lv et al., 2018).

### Statistical analysis

Statistical significance was determined using Student’s *t*-test. All analyses were performed with GraphPad Prism software (version 9.5.0, San Diego, CA, USA), and details of the statistical tests are specified in the figure legends.

## Results

### Garcinone C significantly inhibits PRV replication

**Figure 1:**
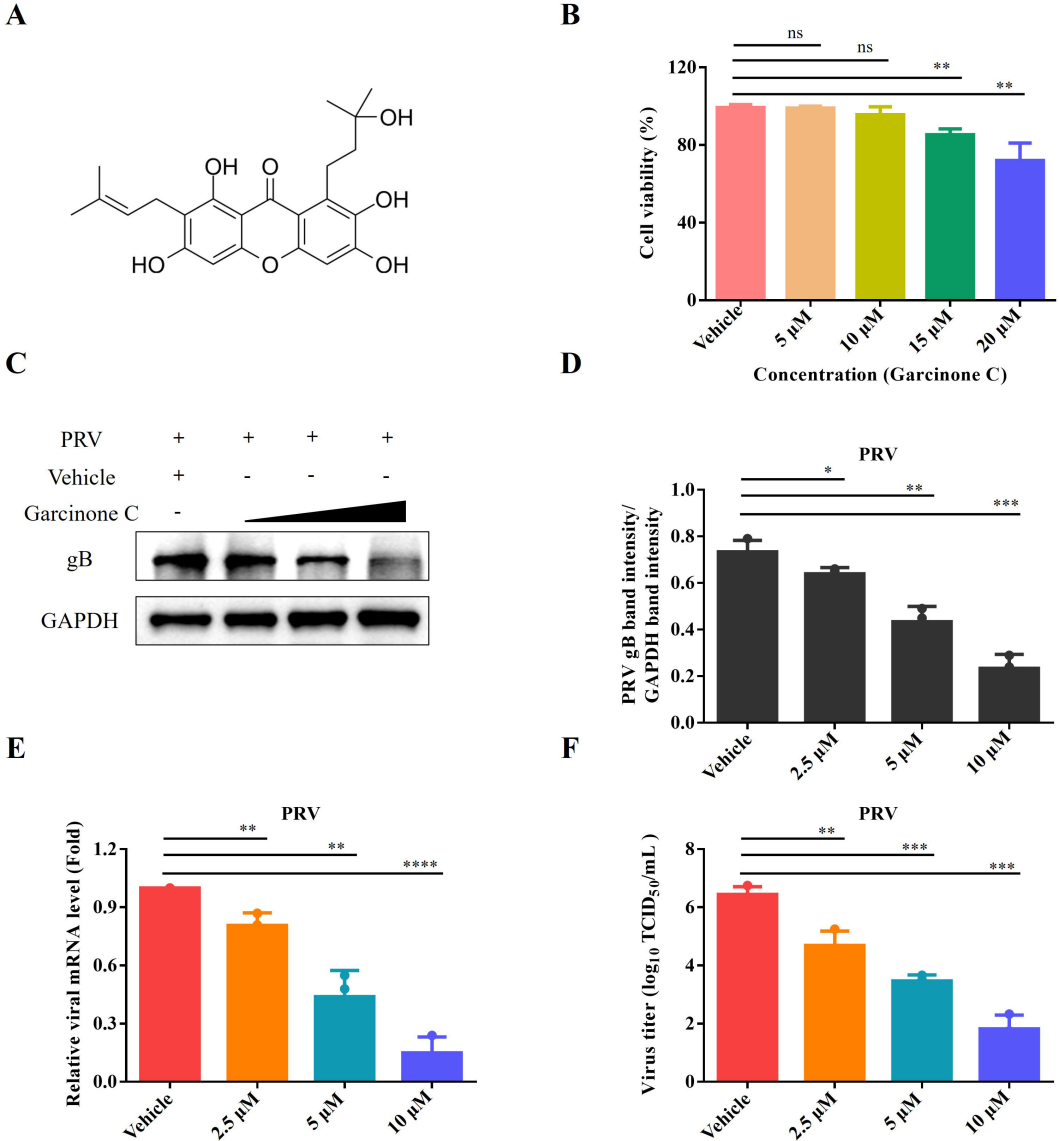
Garcinone C significantly inhibited PRV proliferation *in vitro*. **(A)** Chemical structure of garcinone **(B, C)** Cytotoxic effects of garcinone C on PK-15 cells were assessed using CCK-8 assays after 24 (h) **(C)** The antiviral effect of garcinone C (2.5, 5, 10 μM) against 0.05 multiplicity of infection (MOI) PRV in PK-15 cells was analyzed by Western blotting at 24 hours post infection (hpi). **(D)** The band intensity of PRV gB was quantified using ImageJ software. **(E, F)** The gB mRNA levels and viral titer of PRV in PK-15 cells were detected by qPCR and TCID_50_ assay, respectively (*P < 0.05, **P < 0.01, ***P < 0.001, ****P < 0.0001, ns: non-significant).

The chemical structure of garcinone C was shown in **Figure 1A**. The cytotoxicity of garcinone C for PK-15 cells was evaluated using a CCK-8 assay. The compound was determined to be non-cytotoxic at concentration below 10 μM (**Figure 1B**). Hence, garcinone C at concentration of 2.5, 5, and 10 μM was selected to evaluate anti-PRV activity. Western blotting analysis revealed that expression level of PRV gB protein decreased in a dose-dependent manner upon garcinone C treatment (**Figure 1C**). Quantitative analysis of the gB protein using imageJ software revealed a significant reduction in the garcinone C-treated group compared to the vehicle-treated control (**Figure 1D**). Meanwhile, the mRNA level and viral titers of PRV exhibited a significant reduction in a dose-dependent manner with increasing concentrations of garcinone C (**Figures 1E, F**).

**Figure 2:**
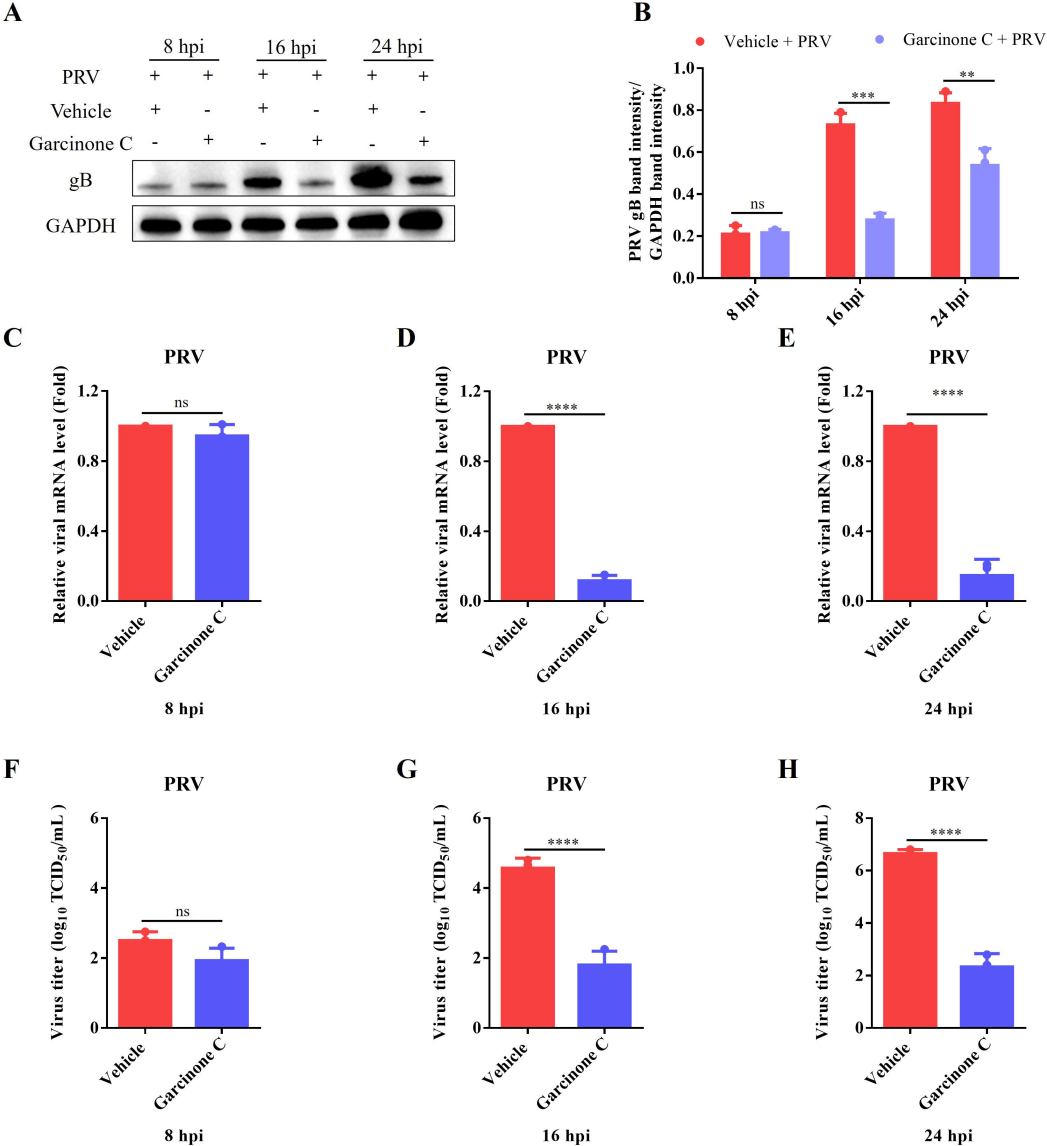
Garcinone C restrained PRV replication in a time-dependent manner. **(A)** PK-15 cells were infected with 0.05 MOI PRV and treated with either garcinone C or vehicle. The gB protein levels were assessed by Western blotting at 8, 16 and 24 hpi. **(B)** Quantification of gB band intensity was performed using ImageJ software. **(C-E)** The gB mRNA levels of PRV were quantified by qPCR at 8, 16 and 24 hpi. **(F-H)** The viral titer of PRV was determined by TCID_50_ assay at 8, 16 and 24 hpi (**P < 0.01, ***P < 0.001, ****P < 0.0001, ns: non-significant).

The antiviral function of garcinone C was further evaluated at different time points after PRV infection. The results of Western blotting displayed viral gB proteins significantly lower in garcinone C-treated cells, compared with those in vehicle control cells at 8, 16, and 24 hpi (**Figures 2A, B**). In line with this, the mRNA level and viral titers were significantly reduced in garcinone C-treated cells relative to vehicle control (**Figures 2C–H**). Furthermore, the half-maximal inhibitory concentration (IC_50_) of garcinone C against PRV infection was evaluated and determined to be 5.081 μM (**Supplementary Figure S1**). Meanwhile, we found that garcinone C also suppressed influenza A virus H3N2 replication (**Supplementary Figure S2**). These results demonstrate that garcinone C markedly restricts PRV proliferation at non-cytotoxic concentrations, and showed broad-spectrum antiviral activity.

### Garcinone C exerts anti-PRV activity in the post-treatment stage

**Figure 3:**
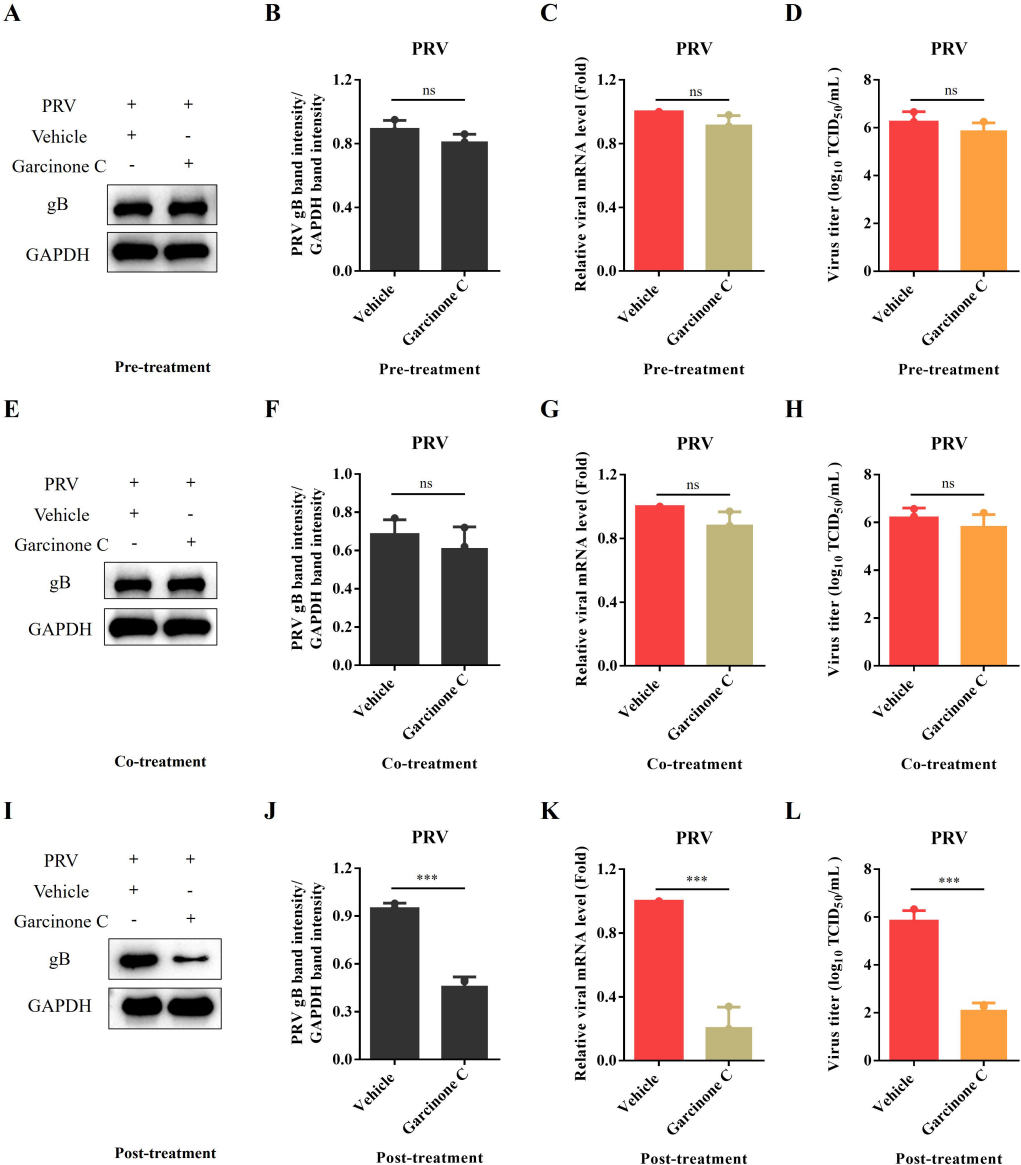
Garcinone C inhibited PRV proliferation at post-treatment stage. **(A, E, I)** Western blotting was used to analyze the antiviral effect of garcinone C under three treatment conditions including pre-treatment, co-treatment, and post-treatment. PK-15 cells were infected with 0.05 MOI PRV, and samples were harvested at 24 hpi for analysis. **(B, F, J)** PRV gB band intensity was quantified by ImageJ under pre-treatment, co-treatment, and post-treatment conditions, respectively. **(C, G, K)** The levels of PRV gB mRNA were quantified by qPCR under three treatment conditions: pre-treatment, co-treatment, and post-treatment. **(D, H, L)** The viral titer of PRV was determined by TCID_50_ assay under three treatment conditions (***P < 0.001, ns: non-significant).

To pinpoint the specific stage at which garcinone C repressed PRV replication, a time-of-addition assay was performed. Cells were treated with the compound at 1 h before (pre-treatment), during (co-treatment), or 1 h after (post-treatment) PRV inoculation. As shown in **Figures 3A–H**, the mRNA and protein levels of gB, as well as PRV titers, were not significantly reduced when garcinone C was administered at pre-treatment or co-treatment stage, compared to the vehicle-treated group. In contrast, post-treatment with garcinone C markedly suppressed PRV replication, as demonstrated by significant reductions in gB mRNA, and protein levels, and viral titers (**Figures 3I–L**). These results demonstrate that garcinone C exerts antiviral effect during the post entry phase of PRV.

### GO and KEGG enrichment analyses for differentially expressed genes

**Figure 4:**
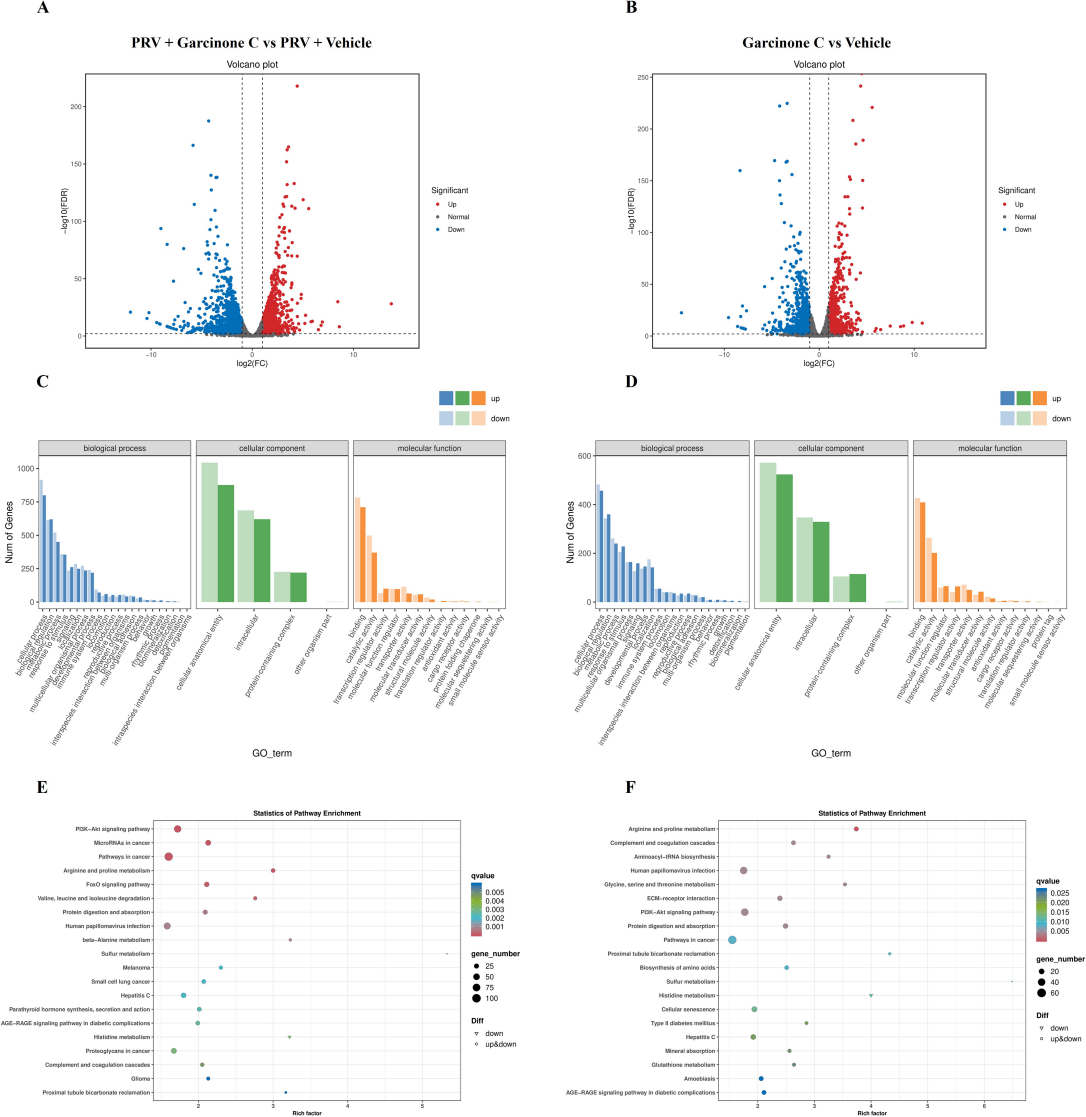
The RNA-seq was performed to identify differentially expressed genes in cells treated with garcinone C after PRV infection. **(A)** The volcano plot displayed differentially expressed genes (DEGs) between the garcinone C-treated and vehicle-treated groups after PRV infection. **(B)** The volcano plot showed DEGs between garcinone C-treated and vehicle-treated groups. **(C, E)** GO and KEGG analyses were conducted on the DEGs identified in the comparison between the garcinone C-treated and vehicle-treated groups after PRV infection. **(D, F)** GO and KEGG analyses were performed on the DEGs identified in the comparison between the garcinone C-treated and vehicle-treated groups.

To elucidate the mechanism by which garcinone C inhibited PRV proliferation, we performed transcriptomic analysis on four experimental groups including vehicle-treated control, garcinone C-treated group, garcinone C-treated after PRV infection, and PRV-infected group treated with vehicle at 24 hpi. Based on (log_2_ |fold change|) > 1 and FDR < 0.01 as the screening criteria, the number of up-regulated differentially expressed genes (DEGs) was 1332, and down-regulated DEGs was 1571 in garcinone C-treated comparing to vehicle-treated after PRV infection group (**Figure 4A**, **Supplementary Table S1**). Between garcinone C-treated and vehicle-treated group, the number of up-regulated DEGs was 768 and down-regulated DEGs was 878 (**Figure 4B**, **Supplementary Table S2**).

Gene Ontology (GO) enrichment analysis of the 2903 DEGs from the comparison between garcinone C- and vehicle-treated groups after PRV infection showed significant enrichment in biological processes, cellular components, and molecular functions. Among them, biological process possessed 22 GO terms, and the most representative were “cellular process” and “biological regulation”. Cellular component possessed 4 GO terms, and the most representative were “cellular anatomical entity” and “intracellular”. Moreover, molecular function possessed 13 GO terms, and the most representative terms were “binding” and “catalytic activity” (**Figure 4C**). Furthermore, the 1646 DEGs from garcinone C-treated and vehicle-treated group were annotated to 38 GO terms. Among them, 21 GO terms represented biological process, and the most representative were “cellular process” and “biological regulation”. The 4 and 13 GO terms belonged to the cellular component and molecular function, respectively, and the most representative cellular component GO terms were “cellular anatomical entity” and “intracellular” while the most representative molecular function GO terms were “binding” and “catalytic activity” (**Figure 4D**).

KEGG pathway enrichment analysis was performed based on the respective DEGs identified from each comparison group. The results showed that the 2903 DEGs were enriched in the pathways in cancer, PI3K-Akt signaling pathway, human papillpomavirus infection, and FoxO signaling pathway (**Figure 4E**). Correspondingly, 1646 DEGs were enriched in pathways in cancer, PI3K-Akt signaling pathway, human papillpomavirus infection, and hepatitis C (**Figure 4F**).

### Common DEGs for GO and KEGG enrichment analysis

**Figure 5:**
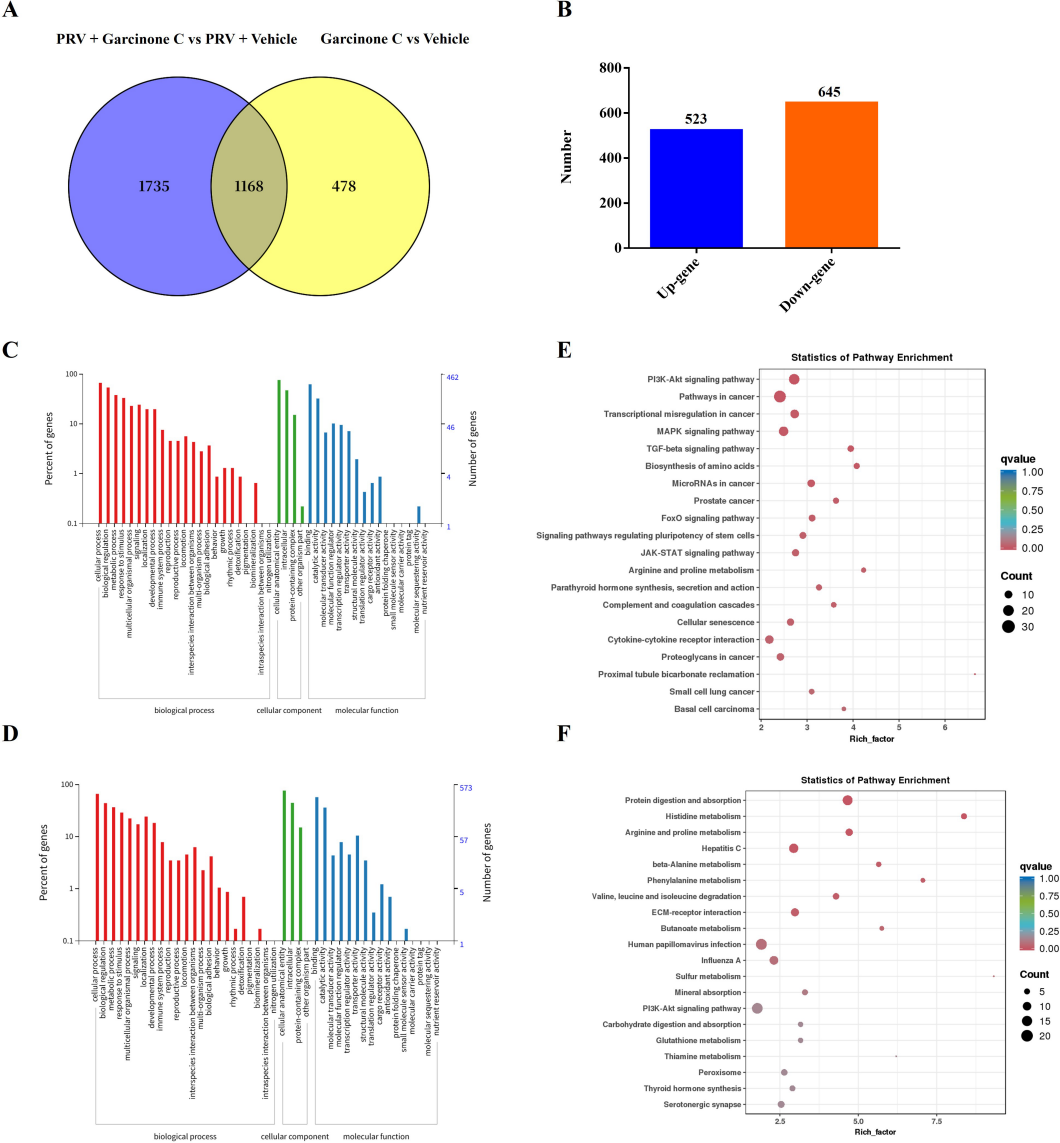
The GO and KEGG analysis of common DEGs. **(A)** The Venn diagram depicted the common DEGs from two comparisons: garcinone C- versus vehicle-treated cells after PRV infection, and garcinone C- versus vehicle-treated mock-infected cells. **(B)** Number of up- and down-regulated genes among the common DEGs. **(C-F)** GO and KEGG analyses were performed on the common DEGs, including both up-regulated and down-regulated genes.

To better explore the role of garcinone C mediated host factors against PRV replication, and narrow the range of targeted host factors for garcinone C. The common DEGs were analyzed between garcinone C-treated versus vehicle-treated after PRV infection group and garcinone C-treated versus vehicle-treated group. As shown in **Figure 5A**, the common DEGs were 1168. Among them, up-regulated common DEGs were 523, and down-regulated DEGs were 645 (**Figure 5B**, **Supplementary Table S3**). The up-regulated and down-regulated common DEGs were annotated to 35 and 34 GO terms, respectively. The most representative GO terms in biological process, cellular component, and molecular function from up-regulated and down-regulated common DEGs were consistent. In the biological process, the most representative GO terms were “cellular process” and “biological regulation”. In cellular component, the most representative were “cellular anatomical entity” and “intracellular”. And the most representative molecular function GO terms were “binding” and “catalytic activity” (**Figures 5C, D**). The KEGG pathway enrichment analysis found that up-regulated common DEGs were enriched in pathways in cancer, PI3K-Akt signaling pathway, MAPK signaling pathway, and JAK-STAT signaling pathway (**Figure 5E**). The down-regulated DEGs were enriched in PI3K-Akt signaling pathway, human papillpomavirus infection, and protein digestion and absorption (**Figure 5F**).

### Garcinone C inhibits the expression of inflammatory cytokines via EGF/PI3K/Akt axis

**Figure 6:**
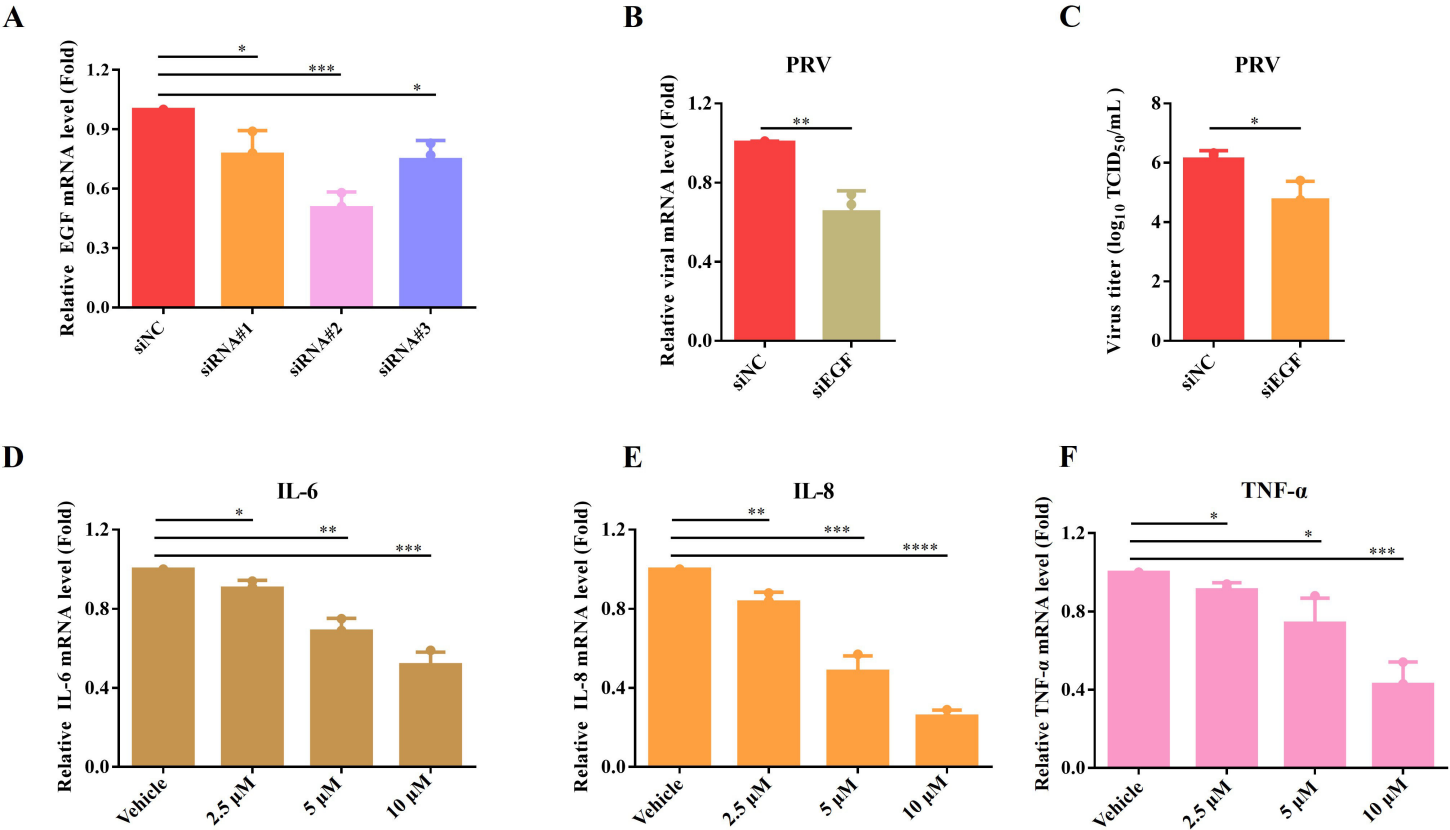
Garcinone C affected the expression of inflammatory factors by modulating EGF. **(A)** Assessment of EGF siRNA knockdown efficiency by qPCR. **(B)** The antiviral effect of EGF knockdown was assessed by qPCR in PK-15 cells infected with 0.05 MOI PRV. **(C)** Viral titer was tested by TCID_50_ assay. **(D-F)** The mRNA levels of IL-6, IL-8, and TNF-α were quantified by qPCR in PK-15 cells following treatment with garcinone C after PRV infection (*P < 0.05, **P < 0.01, ***P < 0.001, ****P < 0.0001).

Garcinone C has been reported to exert anti-inflammatory effects according to previous research (Ji et al., 2024). Meanwhile, the PI3K-Akt signaling pathway mediated the expression of inflammatory factors from KEGG enrichment analysis of common DEGs (**Figures 5E, F**). In order to identify the host factors targeted by garcinone C, we focused more on the PI3K-Akt signaling pathways enriched among the downregulated DEGs. We found that the most significantly downregulated DEGs was EGF (log_2_ |fold change| = 8.4) from garcinone C-treated versus vehicle-treated after PRV infection (**Figure 5F**). It is possible that EGF is the target of garcinone C against PRV infection. To verify this hypothesis, siRNAs for EGF were designed. The siRNA#2 exhibited the highest inhibition effect for mRNA expression of EGF (**Figure 6A**). The siRNA#2 exhibited the highest inhibition of EGF mRNA expression. Compared with the siNC group, viral titers and mRNA levels of PRV in siEGF-treated cells showed significantly reduced (**Figures 6B, C**). Furthermore, the mRNA levels of IL6, IL8, and TNF-α gradually decreased with the increase of garcinone C concentration after PRV infection (**Figures 6D–F**). These results demonstrated that garcinone C targeting EGF reduced the expression of inflammatory cytokines through PI3K-Akt signaling pathway.

### Garcinone C significantly inhibits PRV proliferation in mice

**Figure 7:**
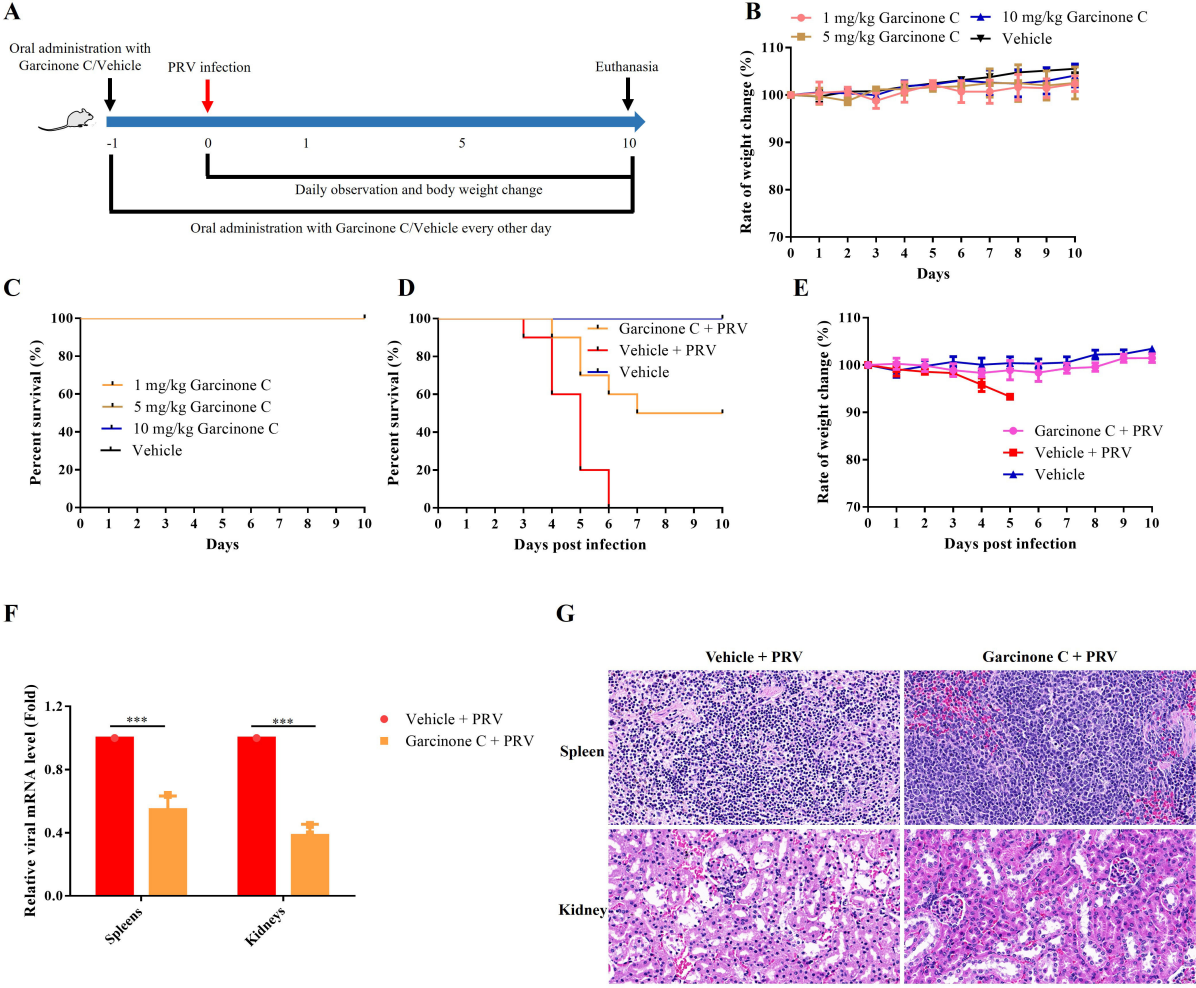
Garcinone C inhibited PRV replication *in vivo*. **(A)** Schematic diagram of the experimental design. Mice were inoculated intraperitoneally with 500 PFU PRV. Garcinone C was orally administered every other day for a total of 6 times, from 1 day before infection to 10 dpi. **(B, C)** Mice were orally administered garcinone C (1, 5, or 10 mg/kg) or vehicle every other day for a total of 5 doses. Body weight and survival were then monitored for 10 days. **(D, E)** Survival and changes in body weight of PRV-infected mice were compared between the group treated with 10 mg/kg garcinone C and the vehicle-treated group. **(F)** Viral mRNA expression levels were quantified by qPCR at 3 dpi. **(G)** H&E staining was performed to analyze histopathological changes in the spleen and kidney of mice at 3 dpi (***P < 0.001). .

To confirm the antiviral effect of garcinone C *in vivo*, we performed an experiment using mice infected with PRV. The experimental scheme was shown in **Figure 7A**. Mice were orally administered 10 mg/kg garcinone C every other day for a total of 6 times, from 1 day before infection to 10 dpi. After taking the garcinone C orally for 1 time, the mice were intraperitoneally injected PRV. The toxicity effect was evaluated by oral administration of garcinone C every other day from day 0 to day 10 for 5 times in mice. Toxicity experiment revealed that oral administration of garcinone C less than 10 mg/kg did not induce significant weight changes and reduce survival rates compared to vehicle-treated control group (**Figures 7B, C**). In addition, the important indicators indicating liver toxicity including aspartate aminotransferase (AST) and alanine aminotransferase (ALT) were detected. There was no significant difference in ALT and AST levels between the drug-treated group and the vehicle-treated group throughout the study, indicating that the drug did not cause liver injury in mice (**Supplementary Figure S3**). The survival rate of mice in garcinone C-treated group was 50% until 10 dpi, while that in vehicle-treated group was full death at 6 dpi (**Figure 7D**). In the garcinone C-treated group, the body weight of mice decreased first and then increased compared to the vehicle-treated group (**Figure 7E**). The viral mRNA levels were reduced in the garcinone C-treated rather than vehicle-treated group (**Figure 7F**). In the vehicle-treated group, the splenic white pulp was disorganized, with a reduced number of loosely arranged lymphocytes. In contrast, garcinone C-treated mice exhibited only mild lymphocyte necrosis and karyorrhexis. Extensive necrosis and detachment of renal tubular epithelial cells were observed, accompanied by nuclear pyknosis and deep staining. However, a small number of tubules exhibited only mild edema (**Figure 7G**). These results indicated that garcinone C inhibited PRV proliferation *in vivo*.

## Discussion

A key strategy for viral replication is manipulating the host’s inflammatory response (Deng et al., 2025). However, an excessive inflammatory response can cause dysregulation of cellular homeostasis, leading to cell death and ultimately impairing the host’s antiviral defense (Li et al., 2023; Wu et al., 2025). PRV infection can also induce a robust inflammatory response in the host (Zhao et al., 2023). Therefore, mediators suppress the expression of inflammatory factors representing a promising approach for antiviral therapy. Garcinone C, a xanthone derivative, can exert anti-inflammatory effects (Ji et al., 2024). We found that garcinone C significantly inhibited PRV proliferation *in vitro* and *in vivo*. Transcriptomic analysis revealed that garcinone C downregulated host factor EGF, and regulated the PI3K-Akt signaling pathway after PRV infection, thereby affecting the expression of inflammatory factors IL-8, IL-6, TNF-α and then exerting antiviral effects.

The viral replication cycle typically comprises essential stages including adsorption, invasion, uncoating, biosynthesis, assembly and release (Xue et al., 2022). We found that, compared to the vehicle control, garcinone C exhibited no effect on viral titers during the pre- and co-treatment stages. Nevertheless, antiviral activity of garcinone C specifically exhibited in the post-treatment stage indicates that it likely targets the biosynthesis phase of the PRV replication. PRV utilizes a large number of host factors to complete self-replication during the biosynthesis process (Strumillo et al., 2021). Hence, host factors have become potential targets for antiviral drugs. Transcriptomic analysis, by quantifying mRNA expression levels, serves as a powerful tool to rapidly and accurately discern differences in host factors (Rao et al., 2021; Ståhl et al., 2016). The gene expression involves transcription followed by translation, which enables transcriptomic to analyze host factors earlier (Woodgate and N, 2023). In the condition, a multitude of upregulated and downregulated DEGs were found in PRV-infected cells treated with garcinone C compared to the vehicle.

The KEGG enrichment analysis of DEGs revealed that garcinone C regulates inflammation- and immune-related signaling pathways after PRV infection. The PI3K-Akt signaling pathway was significantly enriched in the KEGG analysis of our transcriptomic data. The PI3K-Akt pathway plays a pivotal role in essential cellular functions including apoptosis, growth and metabolism, and inflammatory response (Gong et al., 2025; Wang et al., 2025a; Xu et al., 2025). EGF can activate the PI3K-Akt signaling pathway, causing changes in the downstream NF-κB signaling pathway, thereby regulating the expression of host inflammatory factors (Jeong et al., 2014). Previous studies have reported that EGF significantly elevates the expressions of inflammation-related proteins, which demonstrates that EGF plays an important role in the homeostasis of the body (Zhang et al., 2021). Interestingly, garcinone C inhibited PRV proliferation by downregulating EGF expression, which consequently reduced the production of inflammatory cytokines.

Oral administration offers superior convenience and safety characteristic compared to parenteral routes such as intramuscular or intravenous injection, rendering it highly suitable for clinical drug application (Wang et al., 2015; Zhu et al., 2024). We found that oral administration of garcinone C can inhibit the PRV proliferation in mice and improve their survival rate. However, oral administration of garcinone C only achieved a 50% survival rate in mice suggests that its efficacy may be limited by pharmacokinetic profile, such as incomplete intestinal absorption and substantial distributional dilution, which can reduce the effective drug dose reaching systemic circulation and infection sites. Future research may focus on modifying the structure of garcinone C to improve its pharmacokinetic profile and tissue distribution, however, the correlation between structural changes and activity requires further exploration.

In this study, we demonstrated that garcinone C inhibits PRV proliferation *in vitro*, and most importantly, also restricts PRV infection *in vivo*. Transcriptomics analysis revealed that garcinone C reduces the expression of host factor EGF after PRV infection. The down-regulation of EGF suppressed the PI3K-Akt signaling pathway, resulting in diminished production of IL-6, IL-8, and TNF-α. This study provides a foundation for the development of new therapeutic agent against PRV.

## Data Availability

The datasets presented in this study can be found in online repositories. The names of the repository/repositories and accession number(s) can be found below: https://www.ncbi.nlm.nih.gov/, PRJNA1332619.
