# Supplementary material for: Garcinone C inhibits pseudorabies virus replication through EGF/PI3K/Akt axis
Source: Front Cell Infect Microbiol. 2026 Feb 2;15:1722752. doi: 10.3389/fcimb.2025.1722752 (PMC12907380; doi:10.3389/fcimb.2025.1722752)
Supplement: Supplementary file 1 [file Table1.docx]

**Supporting information**

**Garcinone C inhibits pseudorabies virus replication through EGF/PI3K/Akt axis**

Changjie Lv^1,2,3,4^*, Shuang Wang^1,2,3^, Zhongyuan Jin^1,2,3^, Jiaxin Zhang^1^, Yajie Peng^1,2^ and Jinmiao Chen^1,2^

^1^ Key Laboratory of Animal Pathogen Infection and Immunology of Fujian Province, College of Animal Sciences, Fujian Agriculture and Forestry University, Fuzhou 350002, China

^2^ Joint Laboratory of Animal Pathogen Prevention and Control of Fujian-Nepal, College of Animal Sciences, Fujian Agriculture and Forestry University, Fuzhou 350002, China

^3^ Key Laboratory of Fujian-Taiwan Animal Pathogen Biology, College of Animal Sciences, Fujian Agriculture and Forestry University, Fuzhou 350002, China

^4^ Engineering Research Center for Animal Breeding and Sustainable Production, College of Animal Sciences, Fujian Agriculture and Forestry University, Fuzhou 350002, China

*Correspondence:

Changjie Lv, [lvcj0827@126.com](mailto:lvcj0827@126.com)

**
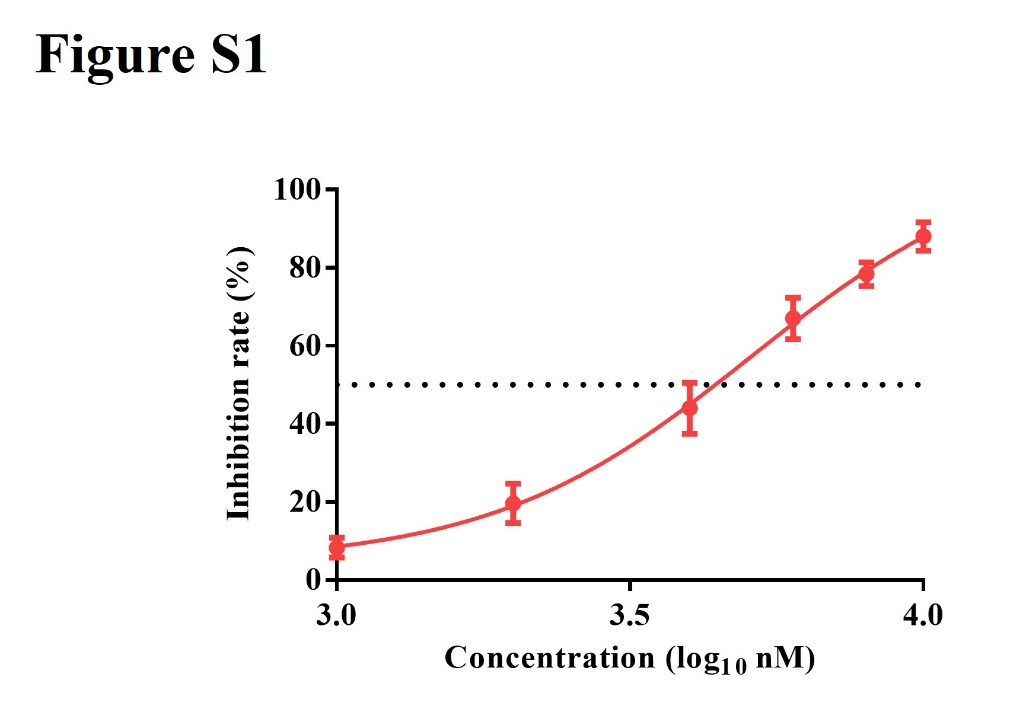
**

**Figure S1. Half-maximal inhibitory concentration (IC_50_) of garcinone C against PRV infection.** Six-point dose response curves were generated with concentrations of garcinone C ranging from 1,000 to 10,000 nmol/L. The cells were infected with 0.05 MOI PRV for 1 h, and then washed and cultured in fresh medium containing garcinone C or DMSO for 24 h. The cells were collected, and the gB mRNA levels of PRV were quantified by qPCR. The inhibition rate was calculated using GraphPad Prism software.

**
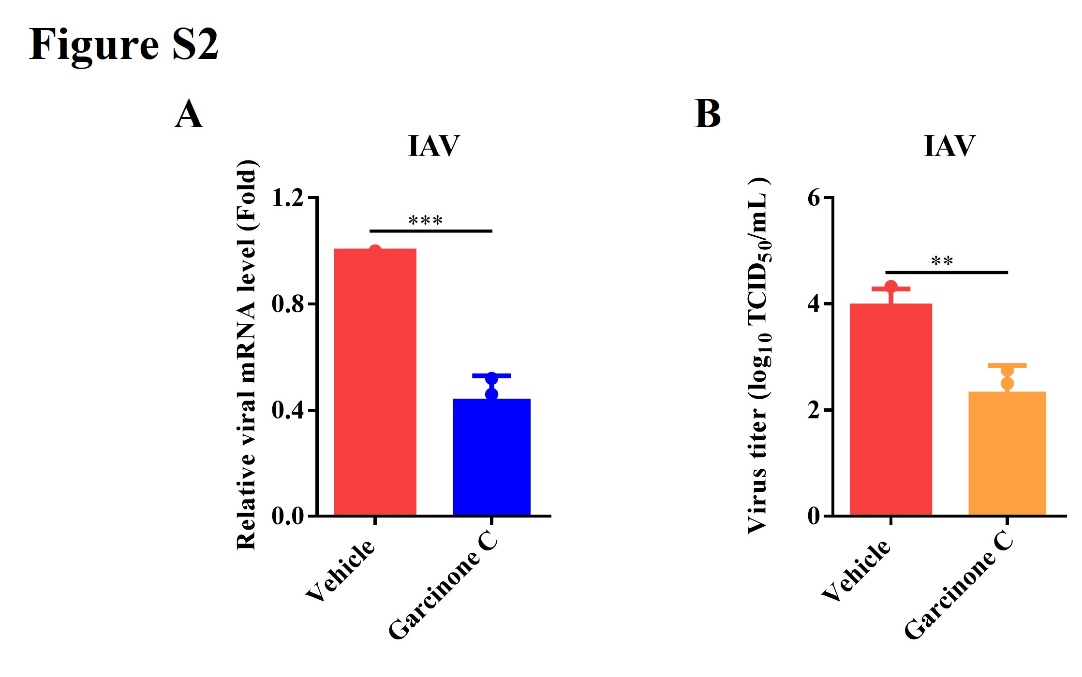
**

**Figure S2.** Garcinone C inhibited IAV H3N2 proliferation *in vitro*. (A) The cells were infected with IAV and treated with garcinone C or vehicle. The mRNA level of IAV was detected by qPCR at 24 hpi. (B) The viral titer of IAV was tested by TCID_50_ assay at 24 hpi (***P* < 0.01, ****P* < 0.001).

**
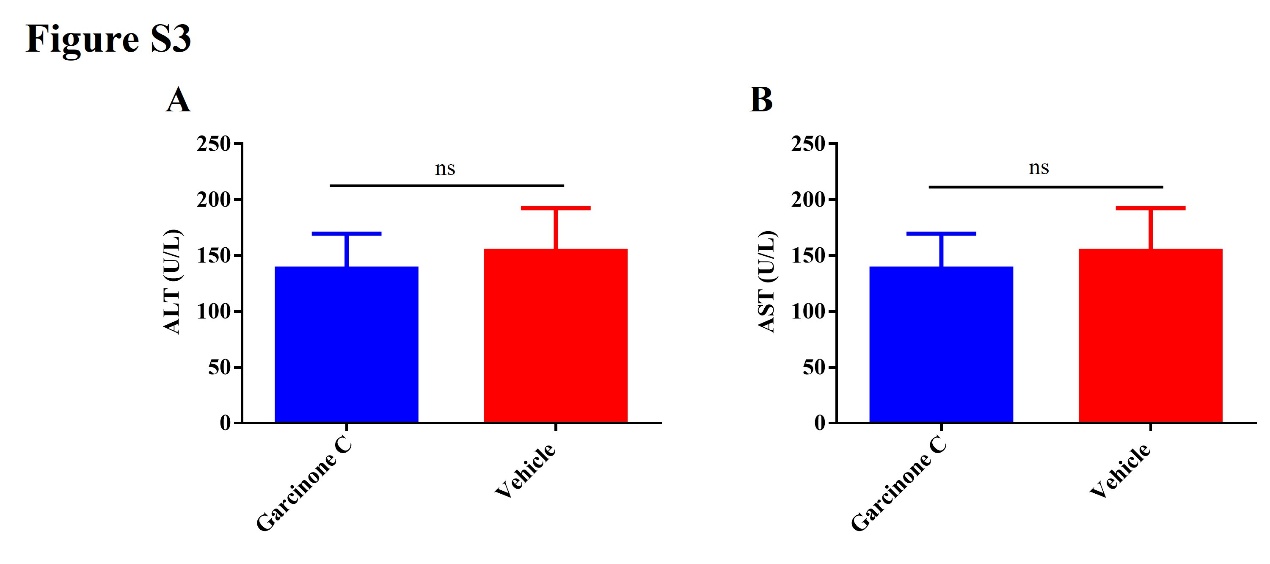
**

**Figure S3.** The liver toxicity effects of garcinone C were assessed. (A) The alanine aminotransferase (ALT) and (B) aspartate aminotransferase (AST) levels were detected in mice compared garcinone C-treated with vehicle-treated group.
